# Type 2 and Non-type 2 Inflammation in the Upper Airways: Cellular and Molecular Alterations in Olfactory Neuroepithelium Cell Populations

**DOI:** 10.1007/s11882-024-01137-x

**Published:** 2024-03-16

**Authors:** Concepció Marin, Isam Alobid, Mauricio López-Chacón, Camilo R. VanStrahlen, Joaquim Mullol

**Affiliations:** 1 INGENIO, IRCE, Fundació Recerca Clínic Barcelona-Institut d’Investigacions Biomèdiques August Pi i Sunyer (FRCB-IDIBAPS), Barcelona, Catalonia Spain; 2 Centre for Biomedical Research in Respiratory Diseases (CIBERES), Health Institute Carlos III, Madrid, Spain; 3 Rhinology Unit and Smell Clinic, ENT Department, Hospital Clínic, Barcelona, Catalonia Spain; 4 Universitat de Barcelona, Barcelona, Spain

**Keywords:** Olfaction, Chronic rhinosinusitis, Post-viral acute rhinosinusitis, Olfactory epithelium neurogenesis, Basal stem cells

## Abstract

**Purpose of Review:**

Neurogenesis occurring in the olfactory epithelium is critical to continuously replace olfactory neurons to maintain olfactory function, but is impaired during chronic type 2 and non-type 2 inflammation of the upper airways. In this review, we describe the neurobiology of olfaction and the olfactory alterations in chronic rhinosinusitis with nasal polyps (type 2 inflammation) and post-viral acute rhinosinusitis (non-type 2 inflammation), highlighting the role of immune response attenuating olfactory neurogenesis as a possibly mechanism for the loss of smell in these diseases.

**Recent Findings:**

Several studies have provided relevant insights into the role of basal stem cells as direct participants in the progression of chronic inflammation identifying a functional switch away from a neuro-regenerative phenotype to one contributing to immune defense, a process that induces a deficient replacement of olfactory neurons. The interaction between olfactory stem cells and immune system might critically underlie ongoing loss of smell in type 2 and non-type 2 inflammatory upper airway diseases.

**Summary:**

In this review, we describe the neurobiology of olfaction and the olfactory alterations in type 2 and non-type 2 inflammatory upper airway diseases, highlighting the role of immune response attenuating olfactory neurogenesis, as a possibly mechanism for the lack of loss of smell recovery.

## Introduction

Despite the remarkable regenerative capacity of olfactory stem cells in the olfactory neuroepithelium, human olfactory deficits are common, especially in the setting of chronic inflammation, remaining their underlying cellular and molecular basis unwell understood.

Upper airway diseases (UAD) encompass a heterogeneous group of pathologies, acute or chronic, whose common pathophysiological process is inflammation of the nasal and paranasal sinus mucosa. Partial (hyposmia) or total (anosmia) loss of smell (LoS) is one of the predominant symptoms in these diseases which has an extraordinary negative impact on the patient’s quality of life, being able to cause depression and put the life of the patient in danger for not detecting odors of dangerous substances [1••]. One of the key mechanisms in the pathophysiology of LoS in inflammatory UAD is the inflammation that occurs in the olfactory mucosa.

Inflammatory responses can be classified into different inflammatory endotypes, the type 2 and the non-type 2 (type 1 and type 3), based on a unique signature profile composed of specific inflammatory mediators, immune cells, and physiologic functions [2]. Type 2 inflammation is characterized by the presence of increased levels of proinflammatory Th2 cytokines as interleukin (IL)-4, IL-5, and IL-13 derived from T-helper 2 (Th2) cells and type 2 innate lymphoid cells (ILC2), alarmins IL-25, IL-33, and thymic stromal lymphopoietin (TSLP) secreted by epithelial cells, as well as recruitment and activation of eosinophils, together with an upregulation of local IgE [3]. Type 1 inflammation is characterized by preferential expression of interferon (IFN)-γ produced by Th1 cells, natural killer cells (NKs), and ILC1 cells [3]. Type 3 inflammation cytokines IL-17 and IL-22 are produced by Th17 cells and ILC3 cells. Neutrophil recruitment, activation, and proliferation occur after type 1 and type 3 responses are activated.

Neurogenesis occurring in the olfactory epithelium is critical to continuously replace olfactory neurons to maintain olfactory function [4, 5••, 6], but is impaired during chronic type 2 and non-type 2 inflammation in the upper airways [7••, 8••]. Several studies have provided relevant insights into the role of basal stem cells as direct participants in the progression of chronic inflammation identifying a functional switch away from a neuro-regenerative phenotype to one contributing to immune defense, a process that induces a deficient replacement of olfactory neurons [8••, 9–13]. The interaction between olfactory stem cells and immune system might critically underlie ongoing LoS in type 2 and non-type 2 inflammatory UAD.

## Olfactory Dysfunction in Type 2 and Non-type 2 Inflammatory UAD

### Chronic Rhinosinusitis

Chronic rhinosinusitis (CRS) is a heterogeneous, persistent, and highly prevalent inflammatory disease of the paranasal sinuses and nasal cavities, with a prevalence of approximately 5–12% in the general population, having a significant impact on quality of life (QoL) [14–16]. The main phenotypes of CRS are with (CRSwNP) or without (CRSsNP) nasal polyps [14]. These two phenotypes have been considered to be characterized by distinct inflammatory endotypes (type 2 and type 1, respectively) [14, 17]; however, recently, it has been considered that inflammation in both CRSwNP and CRSsNP is highly heterogeneous and each phenotype can manifest the three inflammatory endotypes based on the elevation of canonical T-cell cytokines [3, 17].

CRSwNP is the more debilitating of the two phenotypes [14, 15], with patients experiencing a range of symptoms including nasal congestion/obstruction, anterior/posterior nasal discharge, pain/facial pressure, and reduction/loss of smell that persist for > 12 weeks [14]. CRSwNP, with a prevalence of 2–4% in western countries, is a predominantly type 2 inflammation leading to IgE antibody production and recruitment and activation of eosinophils, considered a pathological landmark of CRSwNP [14]. Although CRSwNP is more commonly affecting men, women often experience more severe inflammation and are more likely to have respiratory comorbidities such as N-ERD (non-steroidal anti-inflammatory drug (NSAID)-exacerbated respiratory disease) and asthma [16, 18, 19]. The recurrence rate is more than half in patients of CRSwNP who have significantly higher eosinophilia, particularly in those with > 5% of peripheral blood eosinophils [20, 21].

Olfactory dysfunction is one of the cardinal symptoms of CRSwNP patients with 78% of patients presenting hyposmia or anosmia, with an extraordinary negative impact on the patient’s QoL [1••, 22–24]. Patients with LoS report reduced enjoyment of food potentially lead to eating disorders, failure to smell rotten food, difficulty assessing personal hygiene, depression and social withdrawal, and other comorbidities [25, 26]. LoS is one of the most difficult to treat symptoms in patients with CRSwNP [1••, 22, 27] and correlates with disease severity [28, 29••, 30], being a key predictor of reduced QoL [1••, 31]. Even after endoscopic sinus surgery, 50% of patients still have no improvement in LoS [32].

The pathogenesis of LoS in CRSwNP remains unknown; however, several studies demonstrated that its severity is strongly associated with the expression of type 2 inflammation biomarkers [33, 34, 35••, 36]. In this line, increased eosinophilia in the olfactory mucosa have been correlated with the degree of LoS [37, 38]. Moreover, in CRSwNP patients, elevated levels of Th2-drive proinflammatory cytokines, such as IL-2, IL-5, IL-6, IL-10, IL-13, and IgE in the mucus collected from the olfactory cleft, have been associated with reduced scores for smell test identification [34, 35••, 38].

Although existing studies have greatly improved our understanding of the role of inflammation in LoS in CRSwNP patients, the specific underlying cellular and molecular mechanisms are still not clear.

### Post-viral Acute Rhinosinusitis

Acute rhinosinusitis (ARS) is an acute inflammatory sinonasal disease that almost 100% of the population suffers yearly, with a duration of symptoms < 12 weeks [14, 39], mainly caused by a viral infection (parainfluenza, influenza, rhinovirus, adenovirus, severe acute respiratory syndrome coronavirus 2 (SARS-CoV2, coronavirus disease 2019 (COVID-19)) that can be prolonged on time (post-viral). ARS main symptoms are nasal congestion/blockage/obstruction or rhinorrhea (anterior or post-nasal drip), while the others could be either facial pain/pressure or LoS. The prevalence of ARS in the general population is between 6 and 15%, having a significant impact on QoL [40, 41]. Type 1 inflammation is mainly underlying viral ARS, which is associated with the production of IFN-γ [17, 42••]. Viral infection is one of the most common causes LoS and accounts for 18–45% of all cases [43, 44].

During the first waves of the COVID-19 pandemic, LoS was reported as a frequent clinical sign with a prevalence greater than 47% in the acute phase of the infection [45–48]. LoS caused by other viruses in previous pandemics resulted at a much lower rate [49••, 50], primarily by nasal congestion and obstruction, or by loss of smell as a sequel after the acute infection. However, the rapid onset and severity of LoS in SARS-CoV-2 infection in association with a relative lack of conductive blockade would exclude a conductive pathogenesis [44, 51, 52], suggesting sensorineural underlying mechanisms.

LoS in SARS-CoV-2 infection may occur before, during, or after the occurrence of common symptoms [45]. LoS in SARS-CoV-2 infection has a rapid onset and patients present a severe LoS with relatively nasal congestion [44], which would argue against a conductive pathogenesis [44]. These alterations are often transient; however, a percentage of patients with COVID-19 exhibits LoS that lasts months to years (long COVID-19) [53, 54••, 55, 56]. Recently, it has been described that 5.2% of patients infected during the first wave of COVID-19 and 7.9% of those who had an alteration of smell or taste during the acute phase of the disease remain with symptoms 3 years after COVID-19 [56]. It remains unclear the specific underlying cellular and molecular mechanisms involved in SARS-CoV-2 persistent LoS.

#### Olfactory Neuroepithelium Cell Populations and Neurogenesis

The olfactory neuroepithelium (OE) is a pseudostratified epithelium located in the dorsal part of the nasal cavity. It contacts with volatile odorant entering the nose, an interaction that represents the first step in the transduction process of a given smell. The OE contains three major cells types including bipolar olfactory neurons (ONs), sustentacular cells, and basal cells. Neural stem cells include horizontal (HBCs) and globose (GBCs) basal cells, which reside in the basal layer of OE, possess robust regenerative capacity to replenish ONs lost throughout life to maintain ongoing neurogenesis during adult life [5••, 6, 57].

The sense of smell is mediated in the OE by ONs that detect odorants, transmitting the information to the brain [58]. Bipolar ONS extends a dendrite, which ends in multiple and long specialized cilia in contact with the outside world, being covered with odorant receptors. Moreover, each ON extends a single axon that crosses the cribriform plate reaching the glomerular structure of the olfactory bulb, where they synapse with the second-order neurons that in turn project into the olfactory cortex [59]. Mature ONs are enwrapped by sustentacular cells, which play key roles in odor processing and preservation of epithelial integrity, providing structural and metabolic support to OSNs impacting the way that ONs detect odorants [60–62].

Normal olfactory function depends on cellular regeneration of the OE. The half-life of ONs is of 30 days, and the OE has the ability to regenerate, with normal ONs production through stem basal cells proliferation and differentiation [4, 5••, 6, 63]. GBCs are the main actively proliferating cell population in the OE, being not only responsible for the self-renewal of ONs, but they also are the main cells for regeneration of ONs after minor and selective injury. HBCs are considered to be a reservoir for stem cells that remain inactive in OE homeostasis, being activated by direct and severe epithelial injury and differentiated, mainly into GBCs, which differentiate into almost all epithelial cell types including ONs, and sustentacular cells [5••, 64–67]. All these data suggest a transition in basal cells from unipotent specified differentiation to multipotent state when the OE is subjected to injury [67].

Interestingly, it has been described that supporting/sustentacular cells may affect basal stem cell activation and subsequent OE regeneration [67]. Thus, the ablation of supporting cells but not neuronal depletion in the OE is sufficient for the HBC activation [68, 69]. In this line, it has been shown that the C-X-C chemokine receptor 4 (CXCR4), an essential regulator of olfactory neurogenesis [70], which signaling in tissues depends critically on the levels of its ligand, C-X-C motif chemokine ligand 12 (CXCL12) [71], is regulated by sustentacular cells [72].

Despite the remarkable regenerative capacity of long-lived olfactory stem cells, human olfactory deficits are common, especially in the setting of chronic inflammation, and the cellular and molecular basis remains elusive.

## Effects of Type 2 and Non-type 2 Inflammation on Olfactory Epithelium Cell Populations

### Chronic Rhinosinusitis

In CRS, as result of chronic inflammatory damage and the infiltrating eosinophils, OE loses its normal structure and function [62, 73••, 74]. Eroded OE has been shown to be the most prevalent in CRS patients with anosmia and the highest density of eosinophil infiltration [73••].

In CRS patients with LoS, ONs show alterations in morphology with degeneration of dendrites and/or axons [73••], similar to the ones described in animal models of nasal inflammation induced by lipopolysaccharide [75] or by *Staphylococcus aureus* [76]. These observations suggest that ONs morphologic changes, and the consequent decreased olfactory receptors, may interfere with the normal function of ONs leading to the development of LoS in CRS patients. In addition to morphologic changes, the number of ONs is reduced in CRS patients with LoS [73••, 74]. The number of sustentacular cells has also been shown to be decreased in the injured OE of CRS patients [74, 77], as occurs in animal models of inflammation induced by *S. aureus* [76].

Regarding stem basal cells, their number and function are also affected by type 2 inflammation. In a rodent model of Th2-mediated allergic CRS, a decreased number of immature ONs were found in the OE, in association with increased levels of IL-4, IL-5, and IL-13 and eosinophil infiltration, suggesting a decreased ONs regeneration [7••]. In this line, proliferating HBCs have been found decreased in the OE of CRS patients [8••]. In addition, the ability of HBCs to differentiate is affected since the number of HBCs that are restricted to an undifferentiated state is increased in the OE of CRS patients [8••].

Early research on the mechanisms underlying post-viral LoS consistently shown morphological changes in the OE consisting in a disorganization of the OE architecture with ONs possessing dendrites that did not reach the surface of the epithelium or that were devoid of sensory cilia, reduction in the number of ONs, and respiratory metaplasia [78]. However, the level of reduction in number of neurons does not fully correlate with LoS [79], so further studies have sought to ascertain the effect of viral infection on the function of the remaining ONs.

The high prevalence of LoS after SARS-CoV-2 infection has provided an opportunity to study the underlying pathophysiologic mechanisms of post-viral LoS. Several potential mechanisms for LoS in SARS-CoV-2 infection have been considered, including congestion of the nasal mucosa and obstruction of the olfactory cleft, ONs infection and death, altered ONs function due to cytokine release, immune-mediated downregulation of odorant receptors, and infection and death of sustentacular cells [80–82].

However, several of these mechanisms are now considered improbable [49••] since, for example, the olfactory cleft is not obstructed in most COVID-19 patients with LoS [81, 83, 84]. In addition, LoS in COVID-19 has a sudden start, and, in animal models, it has been shown that a loss of more than 95% of the ONs is required for LoS [85]; however, in anosmic COVID-19 patients, the number of ONs is not reduced to such level [49••, 80, 84]. Regarding the possibility of ONs infection, it is necessary to take in consideration that ONs do not express the virus entry proteins [49••, 80, 81], and although infection of ONs has been suggested in living [86] and deceased COVID-19 patients [87], the fractions of infected ONs were extremely low, being improbable as an underlying mechanism for anosmia [88]. The small number of infected ONs and the lack of evidence of viral replication, due to the very limited neurotropic potential of SARS-CoV-2, suggest that ONs are not the virus main target [78, 79, 82, 86]. Other mechanisms aside from ONs degeneration might be underlying LoS in SARS-CoV-2 infection.

Evidences in experimental models and in human samples have confirmed that sustentacular cells are the main target for SARS-CoV-2 [88, 89••, 90]. Angiotensin-converting enzyme 2 (ACE2) and transmembrane serine protease 2 (TMPRSS2), the entry point for SARS-CoV-2, are expressed in the sustentacular cells being more susceptible than ONs to early infection [89••, 90, 91••]. The lack of ONs support due to the death of infected sustentacular cells may compromise neuronal function leading to LoS in COVID-19 [49••, 92]. It has been suggested that since cell death and regeneration occur much faster in sustentacular cells than in ONs, and thus, this mechanism would be consistent with a sudden LoS in COVID-19, and its rapid recovery in the majority of COVID-19 cases [49••, 80, 91••].

Although the underlying mechanisms of persistent LoS remain less clear, the observation of axonal degeneration in olfactory bulbs and tract tissues in the absence of viral infection in a postmortem study suggests an involvement of a progressive inflammation and immune system activation [93].

## Neurogenesis vs Immune Response in Type 2 and Non-type 2 Inflammatory UAD

**Fig. 1:**
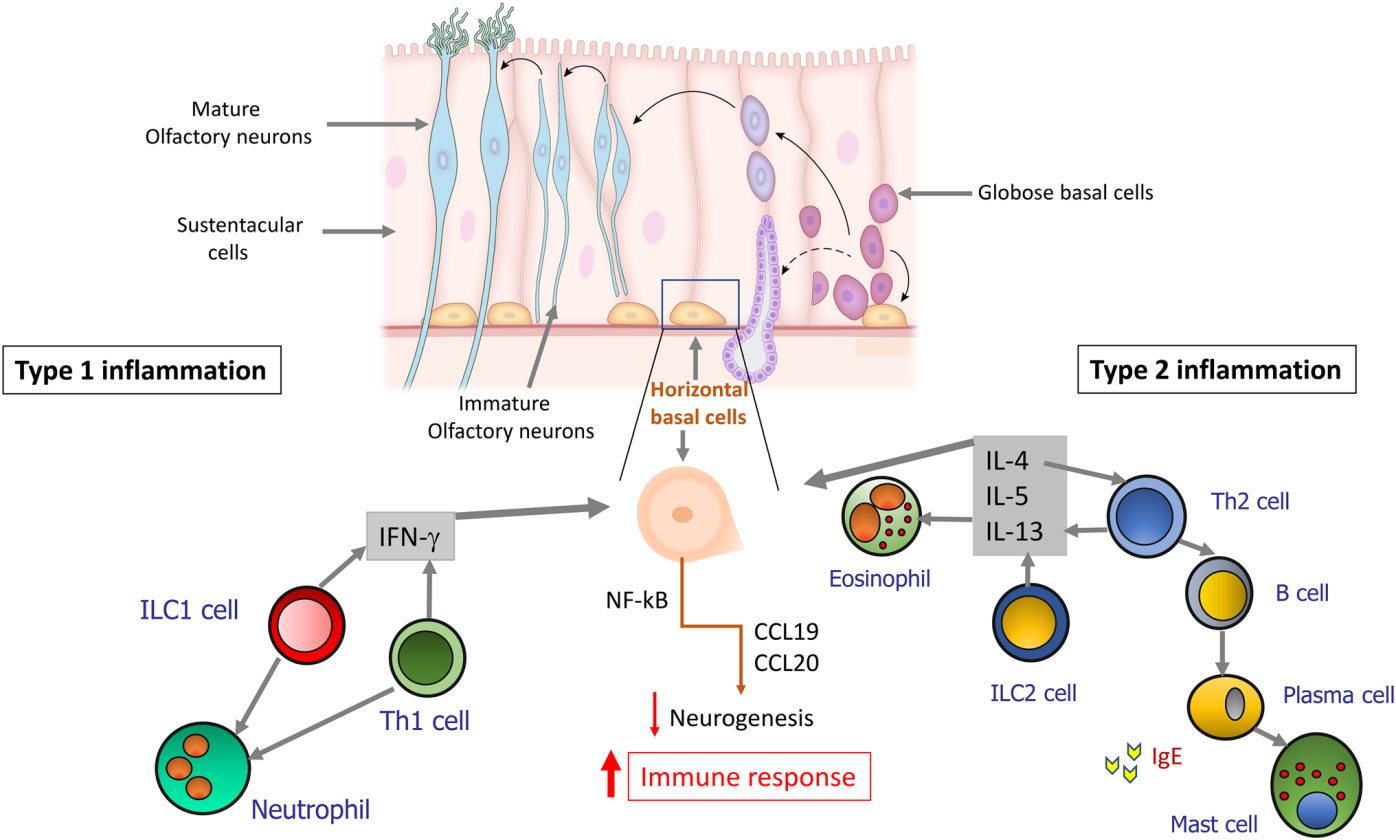
Illustration of how olfactory stem cells control immune response in type 2 and non-type 2 inflammation. In basal conditions, globose basal cells (GBCs) allow olfactory neurons (ONs) replacement differentiating in immature and mature ONs and maintaining olfactory function. After chronic type 2 and non-type 2 inflammation, horizontal basal cells (HBCs) are activated triggering a functional switch from a neuro-regenerative phenotype to one contributing to immune defense

Several studies have provided significant insights into the role of basal stem cells as direct participants in the progression of chronic inflammation and identify a concomitant functional switch away from a neuro-regenerative phenotype to one contributing to immune defense (Fig. 1), a process that induces a deficient replacement of ONs [8••, 9, 13, 17, 49••, 54••, 91••, 94]. This finding suggests that interaction between olfactory stem cells and immune system might critically underlie ongoing LoS in type 2 and non-type 2 inflammatory UAD.

The role of the stem cell–derived chemokines as a potential candidate underlying immune recruitment during inflammation in the olfactory system has been highlighted [8••]. Thus, in an inducible-olfactory inflammation rodent model through the proinflammatory cytokine tumor necrosis factor (TNF) expression from sustentacular cells, the administration of doxycycline resulted in an inflammatory response transforming over the course of weeks an acute phase characterized by cytokine/chemokine induction, neutrophil infiltration, and continuous ONs replacement to an unresolving phase with intense leukocytic (CD45 +) infiltrates and a complete loss of ONs [8••]. Since chronic inflammation resulted in ONs loss, these authors hypothesized that a dysfunction of stem cells may mediate the loss of replacement. They characterized the involved molecular mechanisms observing that both, the TNF and downstream transcription factor NF-κB pathways, which are involved in cell proliferation and immune responses, were enriched in the HBCs, being also upregulated some mediators that regulate immune cell trafficking such as the chemokines CCL19, CCL20, and CXCL19 [8••].

Thus, during chronic inflammation in order to enhance pathogen removal, NF-κB-mediated transcription in HBCs may prioritize immune-related functions rather than proliferation, maintaining the HBCs in an undifferentiated state [8••]. In this line, after doxycycline removal, HBCs underwent under proliferation and differentiation replacing ONs, suggesting that during inflammation, HBCs participate in immune recruitment and modulation, at the expense of ONs replacement [8••]. These authors identified previously unrecognized roles of stem cells in orchestrating immune cell infiltration and local proliferation, elucidating the mechanisms through which HBCs switch off their regenerative function in response to prolonged inflammation. Interestingly, an increase of CD45 + inflammatory cells was observed in the olfactory mucosa form CRS patients consistent with the observation in the genetic model [8••]. The number of immature neurons (tubulin III +) in the OE was slightly increased in CRS patients with moderate inflammation, whereas a decrease in these cells was observed in CRS patients with severe inflammation [8••]. The number of proliferating basal cells in the OE was decreased in CRS patients in association with an increased expression of CCL20 in HBCs [8••]. These observations establish a mechanism of inflammation-associated loss of smell, caused by a functional switch of OE stem cells from regeneration to immune defense and highlight the role of NF-κB in the basal stem cells functional switch, a process which directly blocks neurogenesis, leading to LoS in CRS.

Inflammation after SARS-CoV-2 infection have also been implicated in attenuating neurogenesis [95, 96••]. It is now considered that sustentacular cells may be the main source of cytokines or may contribute among various other cellular sources [83, 84]. Proinflammatory cytokines upregulated during acute and post-acute phases of COVID-19 infection including IL-1β, TNF-α, IL-8, IL-6, IL-15, and the chemokine CCL11 are known to target neural stem cells attenuating neurogenesis [97–101]. Thus, SARS-CoV-2 infection may impair neural stem cell activity resulting in hyposmia/anosmia by impeding olfactory neurogenesis. A link between LoS in COVID-19 and virus-mediated inflammatory disturbance of neurogenesis in the olfactory bulb has been described [102]. Interestingly, the authors associate this deficit with regenerative failure of dopaminergic neurons replenishment in bulbs and OE [102], which are relevant for the maintenance of olfactory function [103]. The impaired olfactory neurogenesis may prevent recovery in the subset of COVID-19 patients with persisting LoS [54••, 96••].

Recently, localized immune cell responses driving phenotypic changes in sustentacular cells and ONs leading to loss of ONs, especially mature neurons, have been described in OE from hyposmic post-COVID-19 patients [54••]. In these patients, severe inflammation appears absent; however, interferon response signatures in the sustentacular cells along with the presence of local lymphocyte populations expressing IFN-γ have been described [55, 91••]. Host immune responses may induce downregulation of genes involved in olfactory signal contributing to the persistence of LoS [49••]. Since chronic immune responses in the OE appear to delay regeneration of the OE [8••], this may explain why a 5% of COVID-19 patients with LoS recover from chemosensory dysfunction late or not at all [49••, 104]. These data provide ongoing mechanistic insights regarding the etiology of SARS-CoV-2-induced long-term LoS.

Understanding the underlying immune mechanisms of the OE in LoS may aid in the development of improved medical treatments for inflammatory type 2 and non-type 2 UAD diseases.

## Conclusions

Olfactory basal stem cells may be directly involved in the progression of chronic inflammation. A functional switch away from a neuro-regenerative phenotype to one contributing to immune defense, a process that induces a deficient replacement of olfactory neurons, has been described in horizontal basal cells. This observation suggests that interaction between olfactory stem cells and immune system might critically underlie persistent loss of smell in type 2 and non-type 2 inflammatory upper airway diseases.

## Data Availability

No datasets were generated or analysed during the current study.
